# Barriers to Office-Based Mental Health Care and Interest in E-Communication With Providers: A Survey Study

**DOI:** 10.2196/mental.6068

**Published:** 2016-08-01

**Authors:** Minnie Rai, Simone N Vigod, Jennifer M Hensel

**Affiliations:** ^1^Women's College Hospital Institute for Health Systems Solutions and Virtual CareToronto, ONCanada; ^2^Department of PsychiatryWomen's College HospitalToronto, ONCanada; ^3^Department of PsychiatryUniversity of TorontoToronto, ONCanada

**Keywords:** e-communication, mental health, technology, barriers, social media

## Abstract

**Background:**

With rising availability and use of Internet and mobile technology in society, the demand and need for its integration into health care is growing. Despite great potential within mental health care and growing uptake, there is still little evidence to guide how these tools should be integrated into traditional care, and for whom.

**Objective:**

To examine factors that might inform how e-communication should be implemented in our local outpatient mental health program, including barriers to traditional office-based care, patient preferences, and patient concerns.

**Methods:**

We conducted a survey in the waiting room of our outpatient mental health program located in an urban, academic ambulatory hospital. The survey assessed (1) age, mobile phone ownership, and general e-communication usage, (2) barriers to attending office-based appointments, (3) preferences for, and interest in, e-communication for mental health care, and (4) concerns about e-communication use for mental health care. We analyzed the data descriptively and examined associations between the presence of barriers, identifying as a social media user, and interest level in e-communication.

**Results:**

Respondents (N=68) were predominantly in the age range of 25-54 years. The rate of mobile phone ownership was 91% (62/68), and 59% (40/68) of respondents identified as social media users. There was very low existing use of e-communication between providers and patients, with high levels of interest endorsed by survey respondents. Respondents expressed an interest in using e-communication with their provider to share updates and get feedback, coordinate care, and get general information. In regression analysis, both a barrier to care and identifying as a social media user were significantly associated with e-communication interest (*P*=.03 and *P*=.003, respectively). E-communication interest was highest among people who both had a barrier to office-based care and were a social media user. Despite high interest, there were also many concerns including privacy and loss of in-person contact.

**Conclusions:**

A high burden of barriers to attending office-based care paired with a high interest in e-communication supports the integration of e-communication within our outpatient services. There may be early adopters to target: those with identified barriers to office-based care and who are active on social media. There is also a need for caution and preservation of existing services for those who choose not to, or cannot, access e-services.

## Introduction

As widespread usage and demand for Internet and mobile technologies grows, a parallel impetus to integrate and shift the traditional modes of health care delivery is emerging. Telemedicine, a long-standing and highly adopted form of remote e-communication in mental health care, has been rated by patients as highly convenient versus in-person consultations [1]. More recently, synchronous videoconferenced care has been extended to be available on personal devices through existing or custom platforms [2,3]. Similarly, software apps installed on mobile phones and other portable devices are being created to assist patients in assessing and monitoring their symptoms or are used as therapeutic tools allowing clinicians to communicate with their patients directly [2]. With a growing number of apps emerging, the use of such mobile technology for mental health care is on the rise. In 2011, of the 9000 consumer health apps that were available, 6% related to mental health, 11% to stress management, 4% to sleep, and 2% to smoking cessation [4]. It is estimated that by 2017, half of the 3.4 billion mobile phone or tablet users across the world will be using some type of mental health care app on their devices [4]. These and other eHealth interventions have the potential to overcome many of the attitudinal and structural barriers associated with accessing mental health care [5], and provide increased convenience for the patient, connect hard-to-reach individuals, reduce stigma, reduce health system costs, and bridge gaps in care provision [2]. The adoption of e-communication tools, which we are defining as online, Internet, or mobile phone-based tools that allow communication between patient and provider, in mental health care is contingent upon effectiveness and the comfort level of patients and providers.

Even among individuals with serious mental illness, mobile phone ownership is 80% or higher [6-9] and there is interest in using mobile apps and e-communication to receive mental health care and monitor symptoms [7,9,10]. The literature suggests that email communication [11,12] and mobile apps [13,14] can be used safely and effectively with a range of mental health patient groups. Of note, younger age groups express more desire and willingness to use technology and may respond to e-communication platforms differently [7]. Furthermore, there are a range of mental health-related services that people may be hoping to access, ranging from information on medication and side effects to reminders for appointments via short message service (SMS) text messaging [3]. The accessibility of e-communication tools for patients, such as having an Internet connection; having a computer, tablet, or mobile phone; having a safe and private place to work; and having adequate experience with using e-communication platforms, are all factors to consider when measuring the appropriateness of mental health therapy integration and technology [3].

Although the potential for e-communication in mental health services is undeniable, it is not entirely without risks, and there has been little formal evaluation of mental health e-communication [3,13]. As a result, it is still unclear exactly how e-communication should be implemented and incorporated into mental health care, and for whom. In this study, we sought to examine factors that might inform how e-communication should be implemented in our local outpatient mental health program. We conducted a survey to examine barriers to traditional office-based care, along with patient preferences and concerns about the use of e-communication tools in their mental health care.

## Methods

### Study Design and Setting

This study was conducted in the Women’s College Hospital Mental Health Program (WMHP), located in downtown urban Toronto, Canada’s largest city. The WMHP provides ambulatory psychiatric consultation and multi-disciplinary treatment in four areas: general psychiatry, mental health in medicine, reproductive life stages, and trauma therapy. The WMHP serves patients of all genders, however, due to the nature of its services, the gender distribution is biased toward women, who comprise about 70-80% of the patient population. The program does not have a catchment and patients are referred from primary care and specialist providers across a wide geographical radius. 

**Figure 1:**
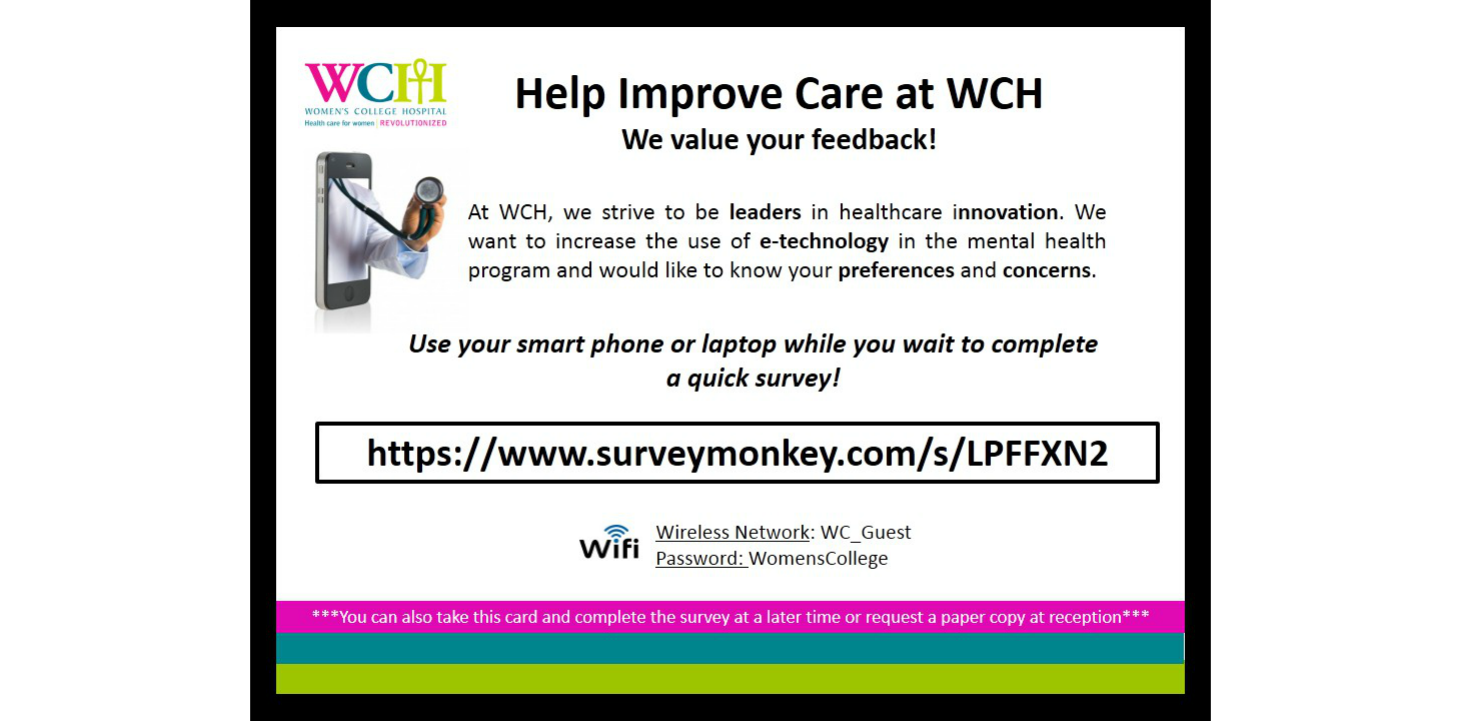
eHealth survey postcard. WCH: Women's College Hospital.

A survey was available in hard copy and as a Web-based survey, hosted by the online survey development cloud-based company, SurveyMonkey. Postcards (see Figure 1) with the survey information and Web address were displayed throughout the waiting room of the WMHP. A free Wi-Fi network is available throughout the hospital. The receptionist also alerted patients to the survey and distributed a hard copy to willing individuals. Patients were free to take the postcards home to complete the survey at a later time. All hard-copy surveys were manually entered into the Web survey and cross-checked for accuracy. Survey participation was entirely voluntary and anonymous, and no personal information was collected. The Web and hard-copy surveys provided a description of the study and details of how data would be used prior to participants completing the questionnaire and, as such, consent was implied with survey completion. The survey was open for 3 months between August and December of 2015. Ethical approval for the study was obtained from Women’s College Hospital Ethics Assessment Process for Quality Improvement Projects.

### Survey Design

The survey was composed of eight items, including both quantitative and qualitative questions in the following topic areas: (1) age, mobile phone ownership, and general e-technology usage (3 items), (2) barriers to attending office-based appointments (1 item); (3) preferences for, and interest in, e-technology for mental health care (2 items), and (4) concerns about e-technology use for mental health care (2 items). We assessed interest and concerns surrounding email and SMS text or Internet messaging; online Web-based assessment tools; mobile phone symptom monitoring apps; health care social networks that may be open to other health care providers, such as family physicians; and personal computer videoconferencing. We asked, in an open-ended question, what respondents would use e-communication to communicate about. The Web survey had one question per page and participants were allowed to review and change their answers throughout the survey. All questions were optional and participation was restricted to one submission per IP address. Prior to the survey launch, a small number of representative individuals completed the survey to ensure usability testing.

### Data Analysis

All survey responses were exported from SurveyMonkey as an Excel file, cleaned, and imported into SPSS version 23 (IBM Corp) for further analysis. We descriptively analyzed all quantitative survey items. We examined the data for associations between the presence of barriers to attending appointments and interest in e-communication, as well as use of social media and interest in e-communication using chi-square statistics. We created a summary *e-communication interest score*, calculated as the sum of responses to questions about the six e-communication tools included in the survey. For the calculation of this score, 2 survey respondents were eliminated because they did not provide a response for any of the e-communication tools questions. If a respondent answered in part, but had a missing response for any item, the score for that item was set to 1 (*not interested*). We created a composite *motivation variable* to represent what we termed the level of motivation to use e-communication. This variable was the interaction between willingness to use based on existing use of social media, and need to use based on the presence of barriers to office-based care. For this variable, we categorized respondents into one of four groups: no barrier, not a social media user; no barrier, social media user; barrier, not a social media user; and barrier, social media user. We compared e-communication interest scores between these groups with the Kruskal-Wallis nonparametric mean rank test, adjusted for multiple comparisons. We also conducted a linear regression with e-communication interest score as the outcome, and age, barrier, and social media use as predictors. Open-ended qualitative survey questions were content analyzed and responses tabulated into thematic categories.

## Results

### Participants

A total of 68 patients completed the survey. Of the 68 respondents, 59 respondents (87%) completed the survey on paper in the waiting room, 3 respondents (4%) completed it in the waiting room on a portable electronic device (eg, tablet or mobile phone), and 5 respondents (7%) completed it at home on a portable device or desktop computer. Table 1 summarizes the demographics, general e-technology use, and barriers to care among respondents. The majority of respondents (51/68, 75%) were between the ages of 25 and 44 years; 91% (62/68) reported owning a mobile phone. A high percentage of respondents reported regularly using SMS text messaging and email, and 59% (40/68) identified as social media users. Just over half (36/68, 53%) identified at least one barrier to attending office-based services, most commonly related to pregnancy, caregiving, work, or distance lived from the hospital. Two or more barriers were reported by 19% (13/68) of respondents.

### Preferences Regarding E-Communication

**Figure 2:**
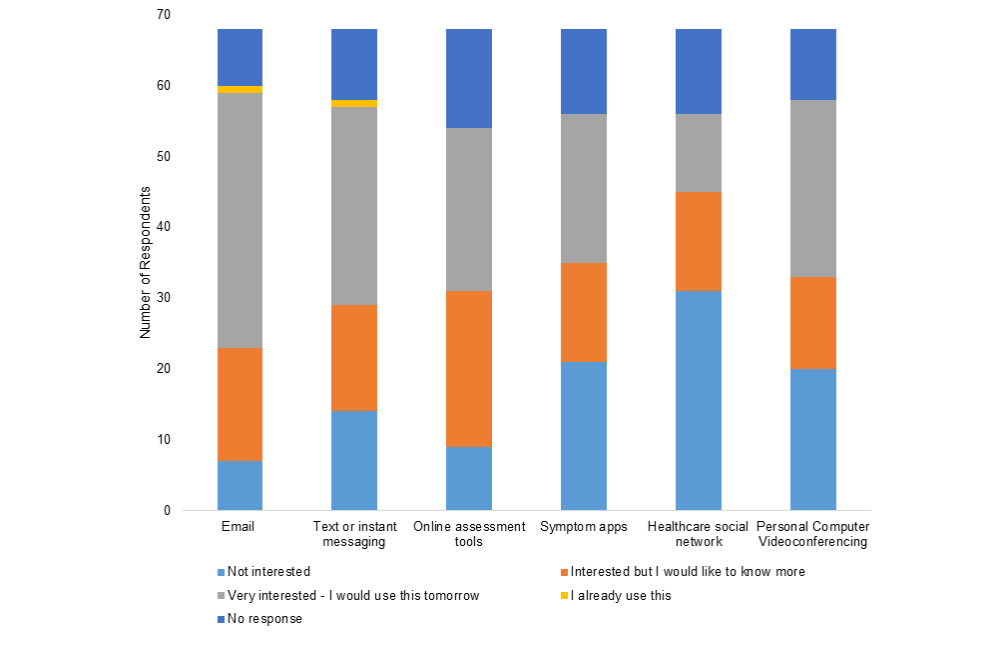
Interest in e-communication for mental health care.

Overall, there was very low existing use of any e-communication between survey respondents and their mental health care providers (see Figure 2). The majority of respondents (52/60, 87%) reported being interested or very interested in using email. Excluding nonresponders on the individual items, more than 60% were interested or very interested in each of SMS text or instant messaging (43/58, 74%), online assessment tools (45/54, 83%), symptom monitoring apps (35/56, 62%), and personal computer videoconferencing (38/58, 66%). Only 44% (25/56) of respondents were equally interested in health care social networks. Compared to respondents with no identified barrier to office-based care, those with at least one were significantly more likely to be interested in online assessment tools (28/30, 93% at least one barrier vs 17/24, 71% no barrier; *P*=.03), a health care social network (18/32, 56% at least one barrier vs 7/24, 29% no barrier; *P*=.04), and videoconferencing (26/34, 76% at least one barrier vs 12/24, 50% no barrier; *P*=.04). Similarly, compared to those who did not, those who identified as a social media user were more likely to be interested in online assessment tools (31/33, 94% social media user vs 14/21, 67% not a social media user; *P*=.01) and health care social networks (21/35, 60% social media user vs 4/21, 19% not a social media user; *P*=.003).

**Table 1:** Demographics, e-communication usage, and barriers to office-based care.

Variable		n (%)
**Age in years (N=67)**		
	Under 25	4 (6)
	25-34	32 (47)
	35-44	19 (28)
	45-54	7 (10)
	55-64	5 (7)
	65+	1 (1)
**Owns a mobile phone (N=68)**		
	Yes	61 (91)
**General e-communication use (N=68)**		
	Email	67 (99)
	SMS^a^ text or instant messaging	57 (85)
	Social media user^b^	40 (59)
**Barriers to care (N=68)^c^**		
	None	36 (53)
	Pregnancy and/or child care	20 (29)
	Work related^d^	11 (16)
	Live too far from hospital	9 (13)
	Medical problems	5 (7)
	Travel related^e^	5 (7)

^a^SMS: short message service.

^b^Facebook, Twitter, LinkedIn, or *other social media site*.

^c^Respondents may have endorsed more than one barrier.

^d^Includes being unwilling/unable to take time off work to come into the office, or workplace being too far from the hospital to do so.

^e^Includes inability to get transportation and/or unwilling to pay for parking.

Analysis of open-ended responses to the question regarding what participants would use e-communication for yielded the following three categories: (1) sharing and feedback, (2) care coordination, and (3) general information. Sharing and feedback captured the desire to share updates on progress with the provider, receive resources such as therapy homework or educational materials, and seek advice, particularly as it related to symptom management and medications. Care coordination related primarily to appointment management, but also to lab requisition and referrals, and information about programming. Three respondents stated they would like to use e-communication for *general information* with no further clarification as to what that would involve.

**Figure 3:**
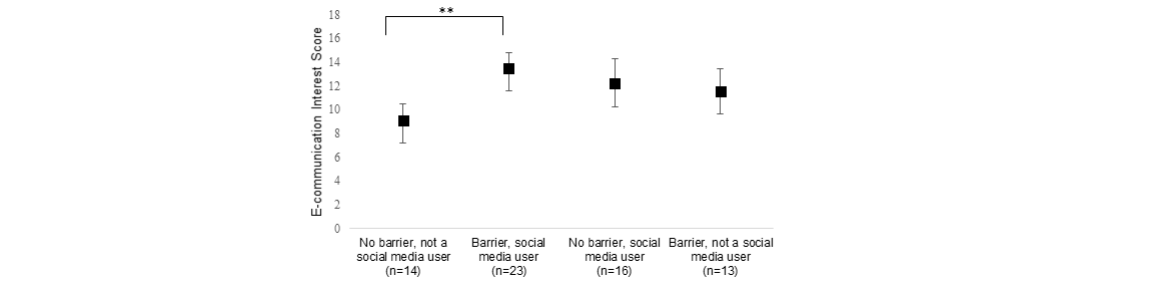
E-communication interest scores across motivation categories. **P=.002 (adjusted for multiple comparisons).

Respondents who identified as a social media user and who had an identified barrier had the highest mean e-communication interest score (see Figure 3), with a significant difference in mean ranks between groups (*P*=.004). The *barrier, social media user* group had a mean rank significantly higher than the *no barrier, not a social media user* group (*P*=.002, adjusted for multiple comparisons) with no significant differences between other groups. The regression model was significant (*F*_3,62_=5.54, *P*=.002, *R*^2^=.21). Both an identified barrier and being a social media user were significantly associated with higher e-communication interest scores (B=1.81, 95% CI 0.23-3.40, *P*=.03; and B=2.51, 95% CI 0.90-4.12, *P*=.003, respectively), each accounting for about half of the explained variance in the outcome.   

### Concerns Regarding E-Communication

Regarding concerns about e-communication, 41 out of 68 respondents (60%) endorsed at least one (see Table 2). A concern about privacy was most commonly reported, followed by already being inundated with e-communication in their lives, and concerns over not having access to the services they prefer. Concerns over loss of face-to-face contact was expressed as an *other* concern by 4 out of 68 respondents (6%). Few respondents did not have access to the Internet or a mobile phone. Interestingly, social media users represented more of the concerns related to privacy (21/32, 66% of privacy concerns). The range of open-ended comments expressed by respondents at the end of the survey varied from a very eager stance to using e-communication tools as a way to connect with their mental health provider, while others reported major concerns about privacy or loss of in-person contact with their provider.

**Table 2:** Concerns surrounding e-communication use for mental health care (N=68).

Variable	n (%)^a^
Privacy	32 (47)
No access to the Internet	2 (3)
No mobile phone	3 (4)
Not interested in signing up for more e-services	4 (6)
I already get too many emails and/or SMS^b^ text messages	8 (12)
If I don’t use the e-communication, I won’t get the same access to services	4 (6)
Other	5 (7)
None indicated	27 (40)

^a^Respondents may have endorsed multiple concerns.

^b^SMS: short message service.

## Discussion

### Principal Findings

Our study found a high level of interest in the use of e-communication for mental health care. The majority of survey respondents already owned and use a mobile phone equipped with downloadable apps, email access, SMS texting functionalities, and built-in cameras for videoconferencing capabilities. In our outpatient population, notable structural barriers to office-based care were present, most commonly being pregnant or having child care responsibilities. Respondents who identified as social media users and had a barrier to office-based care were the most interested in e-communication. While there was an enthusiastic response to the possibility of e-communication, significant concerns about privacy and loss of in-person contact, which for some mental health patients may be particularly meaningful and therapeutic, were reported. A small number of respondents indicated that they would choose not to, or could not engage in, the use of e-communication.

### Comparison With Prior Work

Mobile phone ownership in our study was higher than in other studies. A recent study of adult mental health outpatients in the United States, the majority of whom were from low-income households, found that nearly 80% had a mobile phone [6]. Advanced-feature mobile phone ownership was lower (17%) in that study, however, and our rate was also higher than that found in a survey of an urban emergency department where 50% of patients had an Internet-enabled mobile phone [8]. Advanced-feature mobile phone ownership is rapidly increasing and if these studies were repeated, rates may be higher. Alternatively, our patient population may be of higher socioeconomic status, allowing for higher ownership of the costlier advanced-feature mobile phone compared to the basic-feature phone.

The barriers reported in our sample reflect what is known about the structural barriers to mental health care that are present [5]. Moreover, in a study of postpartum women with depression and pregnancy complications specifically [15], over 60% reported that time was a barrier to seeking treatment for depression. Child care and costs were a barrier for half of the sample. Of note, over 90% of the participants in that study endorsed an interest in Internet-based treatments [15]. Similarly, in a study examining interest in mobile apps for mental health conditions in a more general sample, 67% of patients reported an interest and willingness to try mobile apps designed to monitor their mental health conditions [7]. Most of our sample fell into the age range shown to be most in favor of using e-communication, making the high rates of interest consistent with other age-based assessments [7].

Despite high rates of interest in our sample, rates of existing e-communication between provider and patient in our study were extremely low compared to other studies that have assessed this in other care areas [12]. This may reflect possibly outdated concerns about email use in mental health care [16], as well as practices within our program where emailing patients is discouraged. Beyond email, use of personal videoconferencing for mental health care alone or blended with traditional office-based care [3] is being rapidly adopted, clearly reflecting a patient interest in this type of care delivery as we found in our sample. Our study highlights a high number of patient concerns, despite high interest, that has not been as well represented in the literature on patient preferences. Musiat et al [2] did describe a perception among service recipients that expectations would not be met by computerized treatments versus face-to-face care. We have also described prevalent concerns about privacy and the potential impact on receipt of services for those who may choose not to use or do not have access to e-communication. In addition, our study ties together motivators for e-communication, including barriers to office-based care and use of social media with higher levels of interest for adoption.

### Limitations

This study used a convenience sample of individuals from a mental health program in an urban academic ambulatory hospital that predominantly serves adult female patients, and employs psychiatrists and psychotherapists of varying training backgrounds. We feel that many of our results are generalizable to adult outpatient settings, but our population is unique and the types of barriers endorsed by respondents may not be the same in other programs. Barriers to office-based care, however, are likely to also be prevalent in outpatient clinics that serve a patient population consisting of young parents, and patients engaged in vocational activities. Additionally, the predominant age range in our survey was young and middle-aged adults, so findings may not be generalizable to youth or older adults. We did not collect gender data in our survey, partly because our program is so gender biased toward women, but also because gender has not been shown to be associated with e-technology use or interest in mental health and general health care settings [9,12]. Although we did receive a range of responses, it is possible that some individuals may have been more willing to participate than others. Online survey completion was especially self-guided, whereas hard copies were distributed to some patients by our administrative staff. Although we actually designed this as a Web-based survey, a very small number of respondents completed it online. None of the survey questions were mandatory and, as a result, there were missing data in some questionnaire domains. Finally, the *e-communication score* and *motivation variable* were summary and composite variables, respectively, created by the authors based on the data available and should be interpreted as such.

### Conclusions

E-technology for patient communication is becoming heavily integrated in health care [17,18]. The rapid rate at which mobile technologies are advancing with the potential to link patient and provider [4,13,18] requires some caution, alongside appropriate evaluation and implementation. We have shown that there may be early willing adopters with barriers to office-based care, already actively on social media, who would be good target groups for new e-communication tools to inform implementation more widely. The decision to use e-communication with an individual patient still needs to be part of the treatment assessment and plan. Where barriers to attending office-based care exist, e-communication has the potential to facilitate appropriate follow-up and treatment adherence. It is imperative to address patient concerns and ensure access to equitable services for patients who may not want to utilize e-communication. Assessing and responding to patient concerns about privacy or loss of in-person contact will be based on the type of e-communication being offered and the platform being used. In our institution, as elsewhere, privacy guidelines for the use of e-technology in health care are frequently being updated to align with local policies and legislation. A demonstration of the technology and assurance that traditional care is still available could increase likelihood of uptake among uncertain individuals. With more emerging research to inform recommendations regarding the use of e-communication tools in mental health [3,13], it will hopefully become more clear when, how, and for whom e-communication and mobile technology should be “prescribed.”

## Abbreviations

SMS: short message service
WCH: Women’s College Hospital
WMHP: Women’s College Hospital Mental Health Program
